# Use of Metabolic and Bariatric Surgery Among US Youth

**DOI:** 10.1001/jamapediatrics.2023.0803

**Published:** 2023-05-30

**Authors:** Sarah E. Messiah, Luyu Xie, Nestor de la Cruz-Muñoz, Steven E. Lipshultz

**Affiliations:** 1 University of Texas Health Science Center at Houston School of Public Health—Dallas Campus, Dallas; 2 Dewitt Daughtry Family Department of Surgery, University of Miami Miller School of Medicine, Miami, Florida; 3 Department of Pediatrics, University at Buffalo Jacobs School of Medicine and Biomedical Sciences, Buffalo, New York

## Abstract

This cohort study compares trends in use of metabolic and bariatric surgery among US youth and adults before and after publication of a 2019 American Academy of Pediatrics policy statement on access to such surgery.

Severe obesity (body mass ≥120% of the 95th percentile adjusted for age and sex or an index >35 [calculated as weight in kilograms divided by height in meters squared]) is the fastest-growing obesity subcategory in the US pediatric population.^1^ The severe obesity rate in this population rose from 5.6% in 2015 to 6.5% in 2018, an increase of approximately 4.8 million youths, with the largest increase among Hispanic youth (4.1% in 1999-2000 to 10.7% in 2017-2018), followed by non-Hispanic Black (hereafter Black) youth (from 6.7% to 10.2%, respectively) and non-Hispanic White (hereafter White) youth (from 2.6% to 4%, respectively).^2^ Pediatric obesity is associated with cardiometabolic comorbidities, liver and kidney disease, and lower quality of life, and these associations continue into adulthood.^3^

Behavioral lifestyle interventions alone do not result in long-term, clinically important weight loss among youth with severe obesity.^3^ Metabolic and bariatric surgery (MBS) is a safe and effective treatment.^2,3^ In 2019, an American Academy of Pediatrics (AAP) policy statement highlighted the need for increased adolescent access to MBS when medically indicated.^4^ Recent AAP clinical practice guidelines have supported this policy.^5^ We examined trends in MBS use among US youths aged 10 to 19 years and adults before and after the 2019 AAP statement.

## Methods

Data from merged 2015-2021 participant use files from the Metabolic and Bariatric Surgery Accreditation and Quality Improvement Program (MBSAQIP; asmbs.org/about/mbsaqip) were used. The University of Texas Health Science Center Committee for Human Subjects Protection deemed this cohort study exempt from review and informed consent because it is a retrospective analysis of public, anonymized data sets. This study followed the Strengthening the Reporting of Observational Studies in Epidemiology (STROBE) reporting guideline.

A Cochran-Armitage trend test compared MBS use in 2015-2019 vs 2020-2021 (years before and after the 2019 AAP statement release) in youth aged 10 to 19 years and in adults (aged >19 years) and by racial and ethnic groups (self-reported Black, Hispanic, White, and other [American Indian or Alaska Native, Asian, Native Hawaiian or Other Pacific Islander], multiracial, or unknown or not reported). Statistical analyses were performed using SAS, version 9.4 (SAS Institute, Inc). Two-sided *P* ≤ .05 was considered significant.

## Results

The analysis included 1 346 468 participants (mean [SD] age, 44.9 [11.9] years; 1 101 823 females [81.1%%] and 254 363 males [18.9%]: 227 999 Black [16.9%], 130 601 Hispanic [9.7%], and 773 292 White [57.4%] individuals and 214 576 individuals [15.9%] of other races and ethnicities, multiracial, or unknown race and ethnicity). The MBS completion rates in youths increased from pre-AAP statement release through 2021, overall and for each ethnic subgroup (for all groups, *P* for trend < .001). More youths (n = 1349) (Figure, A) and adults (n = 207 834) (Figure, B) completed MBS in 2021 than in 2020 (1135 youths and 167 119 adults), resulting in 18.85% and 24.36% year-to-year increases in MBS rates, respectively. In 2021, MBS completion increased from 182 to 258 procedures in Black youths, from 179 to 273 procedures in Hispanic youths, and from 459 to 518 procedures in White youths (*P* for trend < .001 for all).

**Figure.**
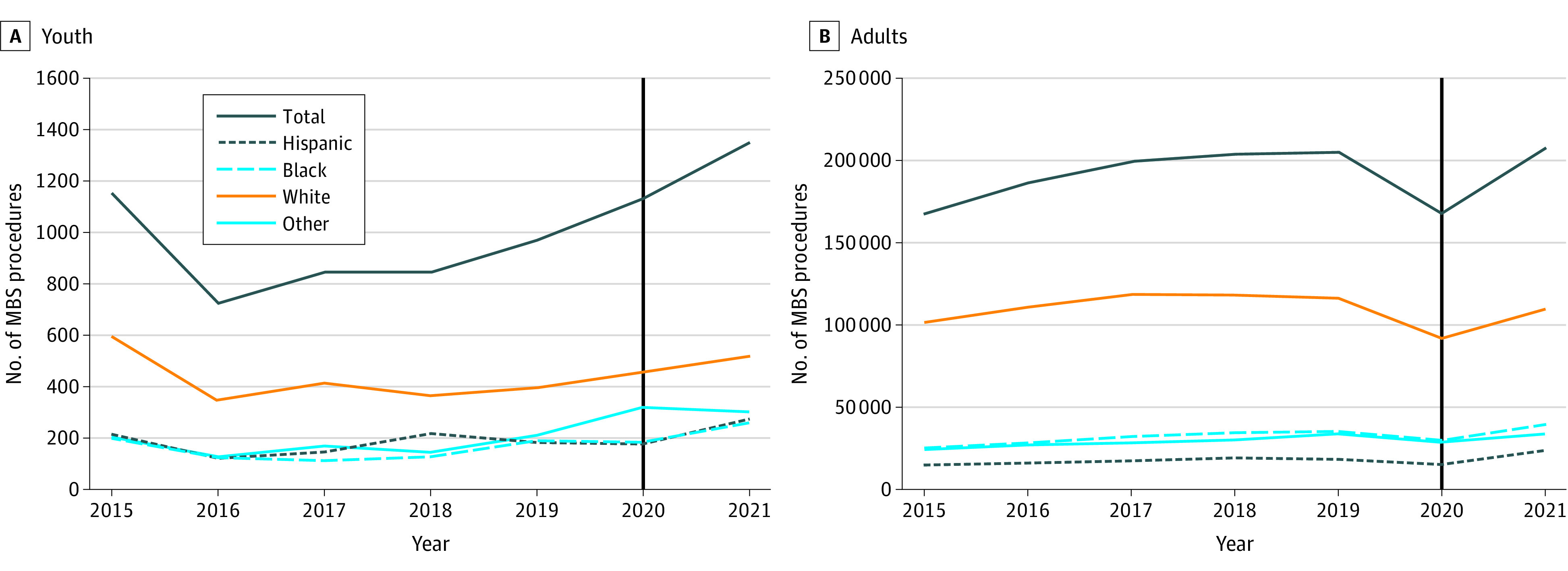
Number of Metabolic and Bariatric Surgery (MBS) Procedures Completed in US From 2015 to 2021 Number of MBS procedures among youth (A) and adults (B) overall and stratified by race and ethnicity before and after (solid vertical line) the 2019 American Academy of Pediatrics statement calling for increased MBS access for adolescents. *P* for trend < .001 in both age groups.

## Discussion

Use of and access to MBS have increased among US youth and among most racial and ethnic groups. Compared with 2015-2019, MBS use in youths increased significantly in 2020-2021 during the first 2 years of the COVID-19 pandemic. In contrast, MBS rates in adults decreased in 2020. The AAP has highlighted the need to educate pediatricians about the benefits of MBS for qualified patients.^4^ Historically, MBS has been underused in youths due to barriers, including low referral rates, limited access, and poor insurance coverage. In a recent study,^6^ our research group reported sustained weight, comorbidity reductions, and low long-term complication rates a decade after MBS in patients aged 15 to 21 years. A study limitation is that the MBSAQIP data may not be representative of all MBS practices in the US. Nevertheless, results of the present study suggest cautious optimism regarding the decreasing barriers to MBS for those US youth in need.

## Notes

Supplement. Data Sharing Statement
