# Supplementary material for: Use of Metabolic and Bariatric Surgery Among US Youth
Source: JAMA Pediatr. 2023 May 30;177(8):856–7. doi: 10.1001/jamapediatrics.2023.0803 (PMC10230368; doi:10.1001/jamapediatrics.2023.0803)
Supplement: Supplement. — Data Sharing Statement [file jamapediatr-e230803-s001.pdf]

Data Sharing Statement

Messiah. Use of Metabolic and Bariatric Surgery Among US Youth. *JAMA Pediatr*. Published May 30, 2023. doi:10.1001/jamapediatrics.2023.0803

Data
Data available: Yes
Data types: Deidentified participant data
How to access data: https://www.facs.org/quality-programs/accreditation-and-verification/metabolic-and-bariatric-surgery-accreditation-and-quality-improvement-program/
When available: With publication

Supporting Documents
Document types: None

Additional Information
Who can access the data: Anyone requesting the data, with permission from the MBSAQIP administrators.
Types of analyses: For any purpose; this data is publicly available.
Mechanisms of data availability: The data will be made available both with and without investigator support. Anyone can get the data from the MBSAQIP website after gaining permission.
